# Epidemiology of childhood constipation: a national study of Swedish children from 2006 to 2023

**DOI:** 10.1186/s12887-025-05858-4

**Published:** 2025-07-03

**Authors:** Helena Kornfält Isberg, Kathy Falkenstein-Hagander, Cecilia Lenander, Mariette Derwig, Lars Hagander

**Affiliations:** 1 Department of Clinical Sciences, Family Medicine and Community Medicine, Lund University, Malmö, Sweden; 2 Unit for Knowledge-Based Management, Department of Health Care Management, County of Scania, Sweden; 3 Pediatric Surgery, Department of Clinical Sciences Lund, Lund University, Skane University Hospital Lund, Lund, 22184 Sweden

**Keywords:** Constipation, Children, Epidemiology, Macrogol, Drugs, Laxative

## Abstract

**Background:**

Childhood constipation is increasingly common, yet its prevalence is uncertain. This study examines comprehensive national data for trends in medically treated childhood constipation over a period of 18 years.

**Methods:**

Included were all Swedish children, 0–14 years, 2006–2023, and all prescriptions for constipation, using the national Swedish Prescribed Drug register. The primary outcome was the number of unique patients prescribed, presented by age, gender, year, county, and ATC-code. Univariate and multivariable linear regression with ecological data were assessed for associations with age, gender, and year of prescription.

**Results:**

The study population increased from 1.54 to 1.84 million children 0–14 years, while constipation increased more than sixfold, from 1.2% in 2006 to 7.9% in 2023. Children 0–4 years were prescribed the most, with an increase from 1.6 to 9.5%. Female gender was associated with 7.5 higher prescription frequency per 1000 inhabitants when adjusted for age and year (4.6–10.5 95%CI, *p* < 0.001). In 2023, girls aged 0–4 years were prescribed 27% more than boys (106.5 vs. 83.7 per 1000), compared with 23% among the 5–9-year-olds (86.8 vs. 70.6 per 1000), and 18% among the 10–14-year-olds (45.0 vs. 38.1 per 1000).

**Conclusions:**

Prescription of constipation drugs to children has increased remarkably during the past 18 years. This study highlights an escalating public health challenge among children, with considerable associated costs for family and society.

## Background

The prevalence of constipation varies between 0.7 and 30% in international studies, representing disparate contexts and definitions [1, 2]. Symptoms related to constipation in children include infrequent painful defecation, often accompanied by abdominal pain and fecal incontinence [3–6]. For this reason, childhood constipation affects quality of life as much as many other chronic conditions in children, and children with constipation have more days from school and more days ‘sick in bed’ or ‘too ill to play’ [7, 8]. With such a burden of disease, high consumption of healthcare services and drug prescriptions can be expected, with high corresponding costs for both society and families [9].

Given the plethora of definitions used in trials and PROM (patient-reported outcome measures) instruments to define constipation, we concentrate our scientific attention to clinically relevant disease in real life by analyzing prescriptions for childhood constipation in a country where the geographic and financial barriers to health-services are but negligible. Previous studies on prescription of medications for constipation are based on limited populations, describe only one group of medications [10], or were performed almost 20 years ago [11]. Those studies describe a significant increase of osmotically active laxatives in children particularly in the younger ages and in females [10, 11]. Overall, however, surprisingly little is known about the magnitude of drug consumption for childhood constipation in a total population of today, and of trends and distributions between and within countries, and if there are clinically relevant differences based on age and gender.

To address these questions, we conducted a comprehensive nation-wide study of prescribed drugs for childhood constipation in Sweden, analyzing the entire Swedish pediatric population aged 0–14 years for trends over an 18-year period of time. The aim of this study was to assess the changes of prescription of constipation drugs in Swedish children over time, stratified according to age, gender, and geographic distribution.

## Methods

This is a national study of all Swedish children, aged 0–14 years, during 2006–2023, monitored in the national Swedish Prescription Drug Register for the occurrence of prescription for constipation. Prospectively reported data to the Swedish National Prescribed Drug Register [12] was retrieved and analyzed retrospectively, and risk factors and changes of prescriptions were described over time. The Swedish National Prescribed Drug Register is hosted by the Swedish National Board of Health and Welfare and provides the basis for official statistics on all prescribed drugs dispensed in pharmacies in Sweden [12].

Primary outcome was the prescription of any drug for constipation, defined as the number of patients prescribed per 1000 inhabitants (and not as the number of prescriptions per 1000 inhabitants). The inclusion of constipation drugs was categorized by WHO ATC-codes [13]. These included Enemas (A06AG), Osmotically acting laxatives (A06AD), Bulk-forming laxatives (A06AC), Contact laxatives (A06AB), as well as Softeners and emollients (A06AA), Peripheral opioid receptor antagonists (A06AH) and Other drugs for constipation (A06AX).

The background population was quantified for age, gender, year of prescription, and county. Pooled ecological data for both primary outcome and the background population categories were publicly available data from 2006 and onwards. Since no individual-level data was collected, analyzed or presented in any stage of the research process, the need for ethics approval and consent to participate was deemed unnecessary according to national regulations under the Swedish law.

The data is presented by age, gender, year of prescription, county, and ATC-code. Age was categorized as 0–4 years, 5–9 years, and 10–14 years. Univariate and multivariable linear regression with ecological data were assessed for associations with age, gender, and year of prescription. P-values < 0.05 were considered significant, and effect estimates were presented as beta with 95% confidence intervals [95%CI]. Statistical software used was IBM SPSS Statistics, version 29.0.2.0.

## Results

**Fig. 1:**
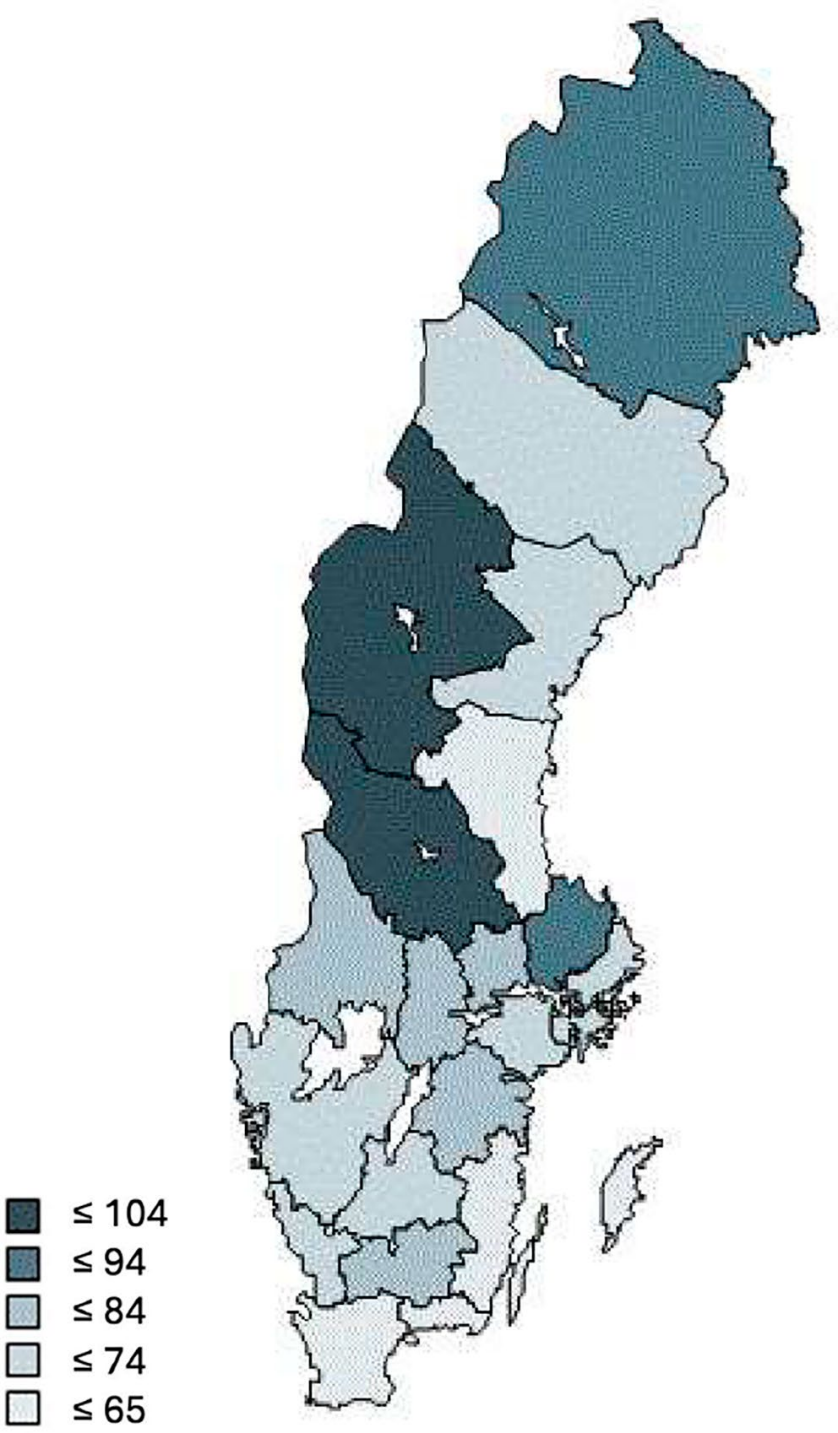
Drugs for constipation in Swedish counties. Patients prescribed per 1000 inhabitants, Swedish children 0–14 years, 2023 [12]

**Fig. 2:**
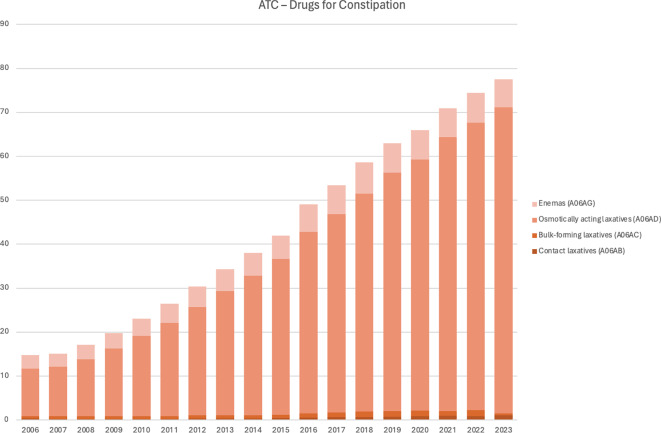
Drugs for constipation by ATC-code. Patients prescribed per 1000 inhabitants, Swedish children 0–14 years, 2006–2023. Enemas (A06AG), Osmotically acting laxatives (A06AD), Bulk-forming laxatives (A06AC), and Contact laxatives (A06AB) are visualized. Softeners and emollients (A06AA), Peripheral opioid receptor antagonists (A06AH) and Other drugs for constipation (A06AX) were so infrequent that they are not visualized [12]

During the study period, the pediatric study population increased from 1.54 to 1.84 million children 0–14 years, with approximately 5% fewer girls than boys. The percentage of children being prescribed drugs for constipation increased more than sixfold, from 1.2% in 2006 to 7.9% in 2023. For each year in the study, the frequency of children being prescribed typically increased by 3.8 per 1000 patients (3.2–4.3 95%CI, *p* < 0.001) (Table 1). There were considerable differences between counties (Fig. 1) and ATC-codes (Fig. 2). Osmotically active laxatives constitute the largest part and the largest increase (Fig. 2).

**Fig. 3:**
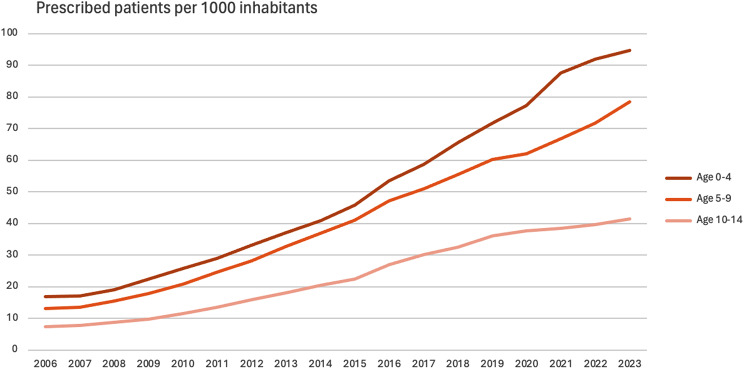
Patients prescribed drugs for constipation per age category, Swedish children, 2006–2023. Prescribed patients per 1000 inhabitants. Ages 0–4 years, 5–9 years, and 10–14 years [12]

Age was negatively associated with drugs for constipation (*p* < 0.001) (Table 1), and in all age groups there was a longitudinal increase in the frequency (Fig. 2). Among children 0–4 years, the frequency increased 5.6-fold from 16.8 per 1000 in 2006 to 94.8 per 1000 in 2023. For children 5–9 years, the frequency increased 6-fold from 13.1 per 1000 in 2006 to 78.5 per 1000 in 2023. For children 10–14 years, the frequency increased 5.5-fold from 7.5 per 1000 in 2006 to 41.5 per 1000 in 2023 (Fig. 3).

**Fig. 4:**
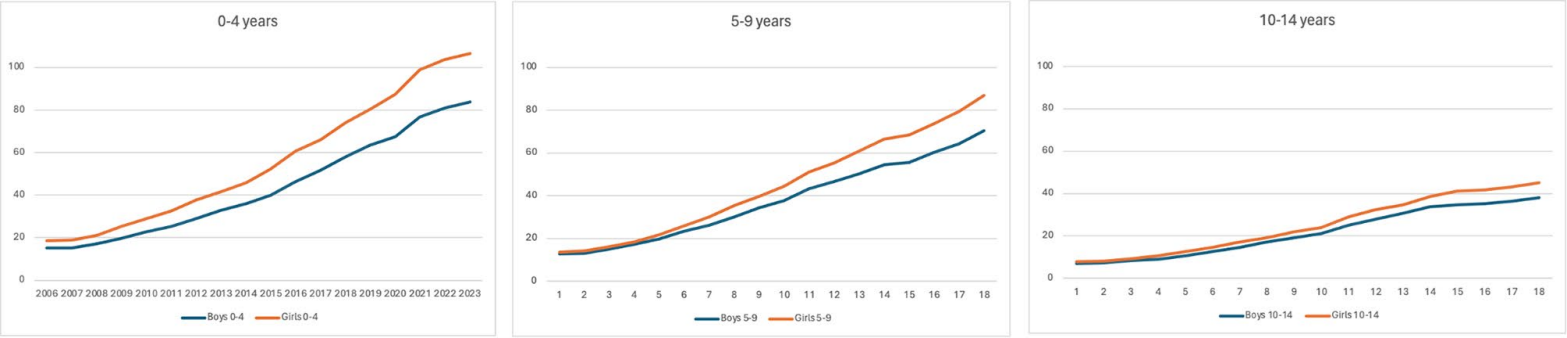
Age category and gender of patients prescribed drugs for constipation Swedish children, 2006–2023. Prescribed patients per 1000 inhabitants. Ages 0–4 years (left), 5–9 years (middle), and 10–14 years (right). Girls (top) vs. Boys (bottom) [12]

Female gender was associated with 7.5 higher prescription frequency per 1000 inhabitants when adjusted for age and year (4.6–10.5 95%CI, *p* < 0.001) (Table 1). The gender difference was largest in the younger age categories (Fig. 4). In 2023, girls aged 0–4 years were prescribed 27% more than boys (106.5 vs. 83.7 per 1000), compared with 23% among the 5–9-year-olds (86.8 vs. 70.6 per 1000), and 18% among the 10–14-year-olds (45.0 vs. 38.1 per 1000) (Fig. 4).

**Table 1:** Patients prescribed drugs for constipation per 1000 Swedish children

	Univariate [95% CI]	*p*-value	Multivariable [95% CI]	*p*-value
**Year** (per year 2006–2023)	3.8 [3.2 to 4.3]	< 0.001	3.7 [3.5 to 4.0]	< 0.001
**Age** (per age category)	-13.1 [-18.1 to -8.1]	< 0.001	-13.1 [-14.9 to -11.3]	< 0.001
**Gender** (Girls vs. boys)	7.5 [-1.5 to 16.6]	0.10	7.5 [4.6 to 10.5]	< 0.001

Patients prescribed drugs for constipation per 1000 Swedish children. Associations per year (2006–2023), age category (0–4 years, 5–9 years, and 10–14 years), and gender (girls vs. boys). Univariate and multivariable linear regression with ecological data. Beta with 95% Confidence Interval (95%CI) and p-value [12].

## Discussion

This study presents the magnitude of medically treated constipation among Swedish children, with trends over 18 years, based on comprehensive national data, stratified by age, gender, and geographical distribution. Our results show that the numbers of children treated for constipation have increased remarkably in all age groups, and that prescriptions for constipation are especially prevalent for girls and younger children. Each year, every 13th child in Sweden has a constipation severe enough to warrant a doctor’s appointment leading to the prescription of drugs.

The Swedish National Prescribed Drug Register was established in July 2005 and contains all prescribed drugs that are dispensed at pharmacies in Sweden. Prescriptions are registered by personal ID number including date of birth and cover the entire population. The register is regulated by different acts and regulations, and pharmacies are required to provide data on prescribed drugs to the register [12]. The data on constipation drugs reported in this study is defined as the number of patients prescribed per 1000 inhabitants and represent patients with at least one prescription of constipation drugs per year. Data on doses per 1000 inhabitant and year was not detected, as this variable will vary according to the size of each prescription, the package size and interval of represcription will differ between prescribers. Also, though being prescribed a drug is not strictly equivalent to having a constipation diagnosis, we believe that for practical purposes it may well approximate the incidence and morbidity, especially since these drugs are exclusively prescribed for the purpose of treating constipation. According to that assumption, we conclude that clinically significant constipation affects 7% (70.82/1000 inhabitants) of all children 0–14 years of age in a country like Sweden, and that the incidence is even higher, 9.5%, in the younger group of children 0–4 years (94.78/1000 inhabitants). This proxy contains risks for both over- and underestimation of the prevalence of constipation diagnosis as prescribed drugs also reflects parents’ care seeking behavior and access to care. It remains unclear if the prevalence of constipation has increased that much. Our study design cannot discern if the rising number of prescriptions rather indicate an overprescribing of drugs or if our findings primarily expose poorly treated constipation in earlier years.

International and Swedish guidelines advocate for lifestyle advice and education as the first line intervention when constipated. If this turns out to be ineffective, drugs are needed for treatment [14]. Macrogol was approved for drug treatment in children with constipation in 2006 [15]. Since then the prescribing of macrogol to children 0–14 years of age has increased 5-fold in Sweden. During this time guidelines for treating constipation changed and the European Society of Pediatric Gastroenterology Hepatology and Nutrition/ NASPGHAN now recommend to integrate early pharmacological treatment with non-pharmacological treatment in young children to interrupt the loop that leads to persistent constipation and to avoid complications such as fecal impaction [16]. Abdominal pain is a common cause for prescribing of macrogol and the side effects are negligible [17]. Causes for the overall increased number of prescriptions, including the age- and gender-specific differences lay beyond the scope of this study.

Prescription data in this study show that constipation is more common in the youngest age category (0–4 years) and in girls which is congruent with other studies [1, 10, 18–21]. A report from the Swedish health agency based on data from the Swedish Prescription Drug Register from 2016 to 2020 in children 0–5 years only including two groups of constipation drugs, report that drug treatment for constipation is more common in girls and that there was an increase in drug treatment for constipation from 5 to 8% during the studied years [21]. In a French study the increased prescribing of macrogol resulted in a simultaneous decrease in the prescribing of enemas [10]. An American study showed that the primary treatment for constipation changed from bulking agents to osmotic laxatives between 1993 and 2004 [22]. In contrast, in our study the prescription of enemas, bulk forming laxatives, contact laxatives and lactulose, previously used drugs, have not declined to an equivalent degree. Previous national and international studies indicate that children with constipation consume more health care [8, 18, 22, 23]. Our study does not have data on ambulatory health care visits for constipation. In an American study based on information from outpatient physician office visits on prescribing constipation drugs the researchers describe a range of non-pharmacological interventions to children depending on region in the US [11]. In a systematic review including studies in adults, the prevalence of functional constipation varied widely between countries, from less than 1% to more than 30% [24]. In our study the prescribing of drugs for constipation varies between counties with the highest prescribing rate in the most north and north-western counties (Fig. 1). We do not know the reason behind the differences in prescribing, but this could be due to different traditions of prescribing among physicians, differences regarding other cultural, environmental and dietary factors among patients. The geographical differences in prescribing could indicate unequal access to healthcare for children. Further studies are needed to elucidate factors that influence the prescribing pattern and the prevalence of constipation in different parts of Sweden.

We could not find any register data on the total number of constipation diagnoses in children from primary health care or pediatric departments in Sweden. In a Swedish prospective study the researchers describe a 14% prevalence of constipation in 12 months old children [23]. In Sweden constipation in children is treated by both primary and pediatric health care services using the same national guidelines [25]. Patients with complicating factors or patients that do not get cured in primary health care are referred to pediatric units. Information on ICD diagnoses from both primary health care and pediatric health care would be valuable to verify the prescribing trend described in this study, and to be able to follow trends in diagnosis over time. The symptoms of constipation are often long lasting and recurring during childhood. Meanwhile no randomized controlled study has evaluated the optimal length of treatment but guidelines suggest a duration of at least 2 months [3]. This study highlights an important public health challenge among children and further research is needed to evaluate whether health care professionals that prescribe treatment for constipation follow national guidelines and why the prescribing of drugs to treat constipation has increased during the last two decades.

This study is congruent with and expands on previous literature, yet the interpretation of our results must be done in the light of our study design. To detect clinically relevant constipation we relied on the judgment of Swedish physicians, yet we have no information on clinical findings, the individual medical history and the indication for the prescription. We believe that the majority of the prescriptions were for constipation since there is no other obvious medical indication. Since no individual-level data was collected we were not able to detect if a child received multiple prescriptions. An increasing frequency of colonoscopy in a country could also contribute to rising numbers, especially if preparations for colonoscopy are done on an outpatient basis. In Sweden, however, this explanatory effect can be expected to be of marginal relevance since the number of colonoscopies per pediatric population is approximately 300 times lower than prescriptions for obstipation, and since the overall frequency of colonoscopy has decreased slightly during the study period while the outpatient frequency has remained constant. Also, we do not know if the patients used the medications once dispensed. A report from the Swedish Public Health Agency describes that prescribing of constipation drugs is more common among children of single parents and if the parents have short education [21], however in this study we do not have any registry data about socioeconomic and life-style factors such as nutrition and physical activity that could contribute to the increasing numbers of constipation and the prescribing of constipation drugs.

## Conclusions

This study shows that prescribed drugs for constipation have increased significantly for children 0–14 years of age during the last 18 years in Sweden. Children 0–4 years and girls are prescribed the most and there are large geographical differences within Sweden. The increase is remarkable, and it is important to contextualize the associated costs for families and society, and to explore underlying factors.

## Data Availability

Data supporting this study are openly available from Socialstyrelsen.se. National Prescribed Drug Register. https://www.socialstyrelsen.se/en/statistics-and-data/registers/national-prescribed-drug-register/.

## Abbreviations

Prom: Patient-reported outcome measures
WHO: World Health Organization
ATC-code: The Anatomical Therapeutic Chemical code
